# THE AGING JOURNEY RAILWAY, ILLICIT DRUG (DE)RAILS, AND CARE: OLDER USERS IN BRAZIL AND PORTUGAL

**DOI:** 10.1093/geroni/igz038.1137

**Published:** 2019-11-08

**Authors:** Maciane Lourenço, Talita Bueno, Priscila Muniz, Robson Pavão, Maria Gabriela França, Jaqueline Da Silva, Emiliane Ferreira

**Affiliations:** 1 Enfermeira do Hospital Federal Servidores do Estado, Federal University of Rio de Janeiro, Brazil; 2 Federal University of Rio de Janeiro, Rio de Janeiro - RJ, Brazil; 3 Federal University of RIo de Janeiro, Federal University of RIo de Janeiro, Brazil; 4 Federal University fo Rio de Janeiro, Rio de Janeiro, Brazil; 5 Hospital Federal Servidores do Estado, Petrópolis, RJ, Brazil; 6 Federal University of Rio de Janeiro / UFRJ - Psychiatry Institute / IPUB, Rio de Janeiro, Rio de Janeiro, Brazil

## Abstract

The study object was motivation to sustain self-care of older drug users. Objective: Analyze older drug users′ motivation to sustain self-care practices. Specific objectives: (i) To characterize social-demographic profile, physical and mental health of older illicit drug users. (ii) Describe everyday practices classified as self-care by older drug users; (iii) Discuss what helps and hinders older drug users′ motivation to sustain self-care. Methodology: Qualitative, descriptive, study using Grounded Theory (GT) to guide the process of data collection, analysis and discussion. Sixty individuals 60+ who used or are current users of illicit drugs were contacted through the snowball technique and interviewed in Brazil and Portugal. Data collection instruments were: (i) a semi-structured question guide; (ii) a spreadsheet for quantitative data collection; (iii) field notes; and (iv) memos were filled out throughout the research process, with the purpose to contribute with and enlighten data analysis and discussion. Results: From data submitted to GT analytical processes, 152 codes emerged, were grouped in 10 subcategories and three categories: “Life trajectory”; “Drugs” and “Health tensions and balance practices”. Conclusion: Data results on older drug users′ motivation to sustain self-care practices gave rise to the central category “the aging journey railway, drug (de)rails and care: ways in Brazil and Portugal” revealing self-care as key in the treatment, also highlighting significant individuals and therapeutic modalities as consubstantial influence for such maintenance.

